# Malaria Resilience in South America: Epidemiology, Vector Biology, and Immunology Insights from the Amazonian International Center of Excellence in Malaria Research Network in Peru and Brazil

**DOI:** 10.4269/ajtmh.22-0127

**Published:** 2022-10-13

**Authors:** Katherine Torres, Marcelo U. Ferreira, Marcia C. Castro, Ananias A. Escalante, Jan E. Conn, Elizabeth Villasis, Maisa da Silva Araujo, Gregorio Almeida, Priscila T. Rodrigues, Rodrigo M. Corder, Anderson R. J. Fernandes, Priscila R. Calil, Winni A. Ladeia, Stefano S. Garcia-Castillo, Joaquin Gomez, Lis Ribeiro do Valle Antonelli, Ricardo T. Gazzinelli, Douglas T. Golenbock, Alejandro Llanos-Cuentas, Dionicia Gamboa, Joseph M. Vinetz

**Affiliations:** ^1^ Institute of Tropical Medicine Alexander von Humboldt, Universidad Peruana Cayetano Heredia, Lima, Peru;; ^2^ Laboratorios de Investigación y Desarrollo, Facultad de Ciencias y Filosofía, Universidad Peruana Cayetano Heredia, Lima, Peru;; ^3^ Department of Parasitology, Institute of Biomedical Sciences, University of São Paulo, Sao Paulo, Brazil;; ^4^ Department of Global Health and Population, Harvard T. H. Chan School of Public Health, Boston, Massachusetts;; ^5^ Department of Biology and Institute for Genomics and Evolutionary Medicine, Temple University, Philadelphia, Pennsylvania;; ^6^ Department of Biomedical Sciences, School of Public Health, University at Albany, State University of New York, Albany, New York;; ^7^ Wadsworth Center, New York State Department of Health, Albany, New York;; ^8^ Instituto de Pesquisas Rene Rachou, Fundação Oswaldo Cruz, Belo Horizonte, Brazil;; ^9^ Division of Infectious Disease and Immunology, Department of Medicine, University of Massachusetts Medical School, Worcester, Massachusetts;; ^10^ Section of Infectious Diseases, Department of Internal Medicine, Yale School of Medicine, New Haven, Connecticut

## Abstract

The 1990s saw the rapid reemergence of malaria in Amazonia, where it remains an important public health priority in South America. The Amazonian International Center of Excellence in Malaria Research (ICEMR) was designed to take a multidisciplinary approach toward identifying novel malaria control and elimination strategies. Based on geographically and epidemiologically distinct sites in the Northeastern Peruvian and Western Brazilian Amazon regions, synergistic projects integrate malaria epidemiology, vector biology, and immunology. The Amazonian ICEMR’s overarching goal is to understand how human behavior and other sociodemographic features of human reservoirs of transmission—predominantly asymptomatically parasitemic people—interact with the major Amazonian malaria vector, *Nyssorhynchus* (formerly *Anopheles*) *darlingi,* and with human immune responses to maintain malaria resilience and continued endemicity in a hypoendemic setting. Here, we will review Amazonian ICEMR’s achievements on the synergies among malaria epidemiology, *Plasmodium-*vector interactions, and immune response, and how those provide a roadmap for further research, and, most importantly, point toward how to achieve malaria control and elimination in the Americas.

## INTRODUCTION

The vast majority of malaria cases currently reported in the Americas occur in the Amazon Basin, which includes nine countries of South America.^1^ Between 2000 and 2020, malaria cases and deaths declined by 58% and 56%, respectively, despite setbacks from a drastic increase in malaria in Venezuela (fewer than 36,000 cases in 2000 and more than 467,000 in 2019).^1^ Although Amazonian malaria cases and associated deaths are a very small portion of the worldwide toll, the social and economic burden is high, Latin American Ministries of Health place a high priority on malaria control and elimination, and the region has unique challenges for control and elimination that require new knowledge and innovative actions.

Deforestation in the Amazon region is directly related to malaria transmission, as shown by investigators of the Amazonian International Center of Excellence in Malaria Research (ICEMR) and others.^2^^–^^9^ Deforestation and other destruction of natural habitat in the Amazon region have increased in the past 5 years, mostly due to illegal exploitation of natural resources.^10^^–^^14^ In addition, illegal gold mining activities have contributed to further forest removal and malaria transmission.^10^^–^^14^ Climatic conditions, also affected by the reduction of the forest cover, have been changing and the number of extreme weather events has intensified, with more than a dozen in the past 25 years—for example, climate patterns in the Pacific Ocean (El Niño and La Niña) and extreme droughts or rains not associated with the Pacific. Human mobility related to mining, other resource exploitation, land settlement, and a network of connections among cities and agricultural areas is intense and contributes to the circulation of parasites with ever-changing dynamic microgeographic introductions and reintroductions. Occupation- and other social factor-related mobility is a particularly important mechanism of maintaining endemic malaria in Amazonia, especially given the high prevalence of asymptomatic malaria infections in the region. All of these factors pose a major challenge for malaria surveillance, diagnosis, control, elimination, and prevention from reintroduction in areas that have achieved elimination. Several areas are of emerging concern driven by cross-border mobility: French Guiana, Suriname, and Brazil, fueled in large part by gold mining,^15^ and Brazil, Guyana, and Venezuela, fueled by gold mining and exacerbated by the continued intense movement of Venezuelans fleeing the country because of political instability.^16^ Newly recognized outdoor-biting behavior by vectors contributes to significant proportions of outdoor malaria transmission, evading standard malaria control measures, such as insecticide-impregnated bednets and indoor residual spraying, leading to residual foci—resilient malaria that requires specific control measures.^17^

With regard to elimination, Brazil launched an elimination plan in 2015 focused on *Plasmodium falciparum*. Recently, it launched a national elimination plan, with four phases: 1) preparation phase, with the goal to reduce the incidence to <68,000 cases by 2025; 2) consolidation phase, with the goal to achieve zero deaths due to malaria, and to eliminate *P. falciparum* transmission by 2030; 3) elimination phase, with the goal of keeping zero deaths, zero *P. falciparum* cases, and to eliminate malaria transmission by 2035; and 4) prevention of reintroduction phase, with the goal to maintain the country free of malaria. As for Peru, in 2017, the government put in place a formal malaria elimination plan (2017–2021), the Malaria Zero Program (MZP), that takes a community-level approach to control malaria with the goal of elimination by 2030 with three phases: 1) the control phase, testing and treating, which focuses on the elimination of symptomatic infections and has a duration of 3 years; 2) a elimination phase, the goal of which is to eliminate malaria parasites from individuals at a regional level by targeting asymptomatic and low parasite density infections; and 3) a final elimination phase, to identify and ameliorate residual malaria transmission foci, including reintroductions.^18^ The MZP was successful in the first (control) phase prioritized in the high endemicity region of Loreto decreasing the number of cases by 74.5% from 2017 to 2021. A second malaria elimination plan, which describes in detail the elimination phase involving not only the Loreto region but also the whole country, was published in 2022.^19^

Malaria transmission in Amazonia, as elsewhere, takes place in a dynamic, complex, and constantly evolving context, where malaria remains resilient despite standard control measures. In that context, and to shed light on the biological and sociodemographic challenges to malaria elimination, the ICEMR network in Peru and Brazil was established.

## ORIGINS AND GOALS OF THE AMAZONIAN INTERNATIONAL CENTER OF EXCELLENCE IN MALARIA RESEARCH

Field-based fundamental malaria research in Peru became active with US National Institute of Allergy and Infectious Diseases (NIAID) funding in the mid-2000s and focused on identifying human reservoirs of malaria transmission. The high prevalence of asymptomatic *Plasmodium* parasitemia (as high as 50%) became an organizing principle for malaria field research in Amazonia, where it was recognized that understanding the combined sociodemographic and immunological factors in maintaining endemic, resilient malaria was key to developing new approaches to future malaria control.^20^ The first high impact reports from Rondônia, Brazil, demonstrated that asymptomatic *Plasmodium* infections were detected in 20–50% of the study participants by molecular methods and were four to five times more frequent than full-blown clinical malaria;^21^^–^^23^ similar findings were reported from Iquitos, Peru.^24^ Amazonian ICEMR studies carried out in the same settings in 2015 confirmed these earlier findings: quantitative PCR demonstrated a geometric mean *Plasmodium vivax* parasitemia of 90 parasites/µL, (95% CI [42–190]) with 50% of subjects having fewer than 10 parasites/µL.^25^

These observations provided the major rationale to formally create a field-based malaria research platform in the Peruvian Amazon. Such a platform has been instrumental to others, such as the pivotal clinical trials of tafenoquine.^26^^–^^29^ The Iquitos region of the Loreto Department of Peru has had a long-standing NIAID-supported malaria research program focused on understanding the dynamics of *P. vivax*, explicitly examining the contribution of the asymptomatic human reservoir to continuing malaria transmission.^30^ As part of such efforts, the need to improve entomological and more broadly, vector biology information and tools was clear. An important achievement was establishing the first reported long-term colony of *Nyssorhynchus *(formerly *Anopheles*)* darlingi*, originating from wild-caught mosquitoes in Loreto, in Iquitos,^31^ Peru, where this important malaria vector has been continuously colonized for more than a decade. Based on the Peruvian experience, transfer of know-how led to similar success in establishing a long-term colony of *N. darlingi* in Rôndonia State, Brazil, also originating from local wild-caught mosquitoes.^32^^,^^33^ After carrying out experimental *Ny. darlingi* with F1 mosquitoes using *P. vivax* obtained ex vivo from infected humans, 30 being able to produce consistent quantities of *N. darlingi* has enabled field-based transmission studies of experimental mosquito infections well as production of *P. vivax* sporozoites for experimental work.^31^^,^^32^^,^^34^^–^^36^

NIAID-supported field-based malaria research in Brazil started in 2004. At that time, hypoendemic malaria transmission prevailing across the Amazon Basin was assumed to rarely elicit the status of clinical immunity seen among adults exposed to holoendemic malaria in Sub-Saharan Africa,^37^^,^^38^ except for remote riverine populations that are continuously exposed to infection since birth. This hypothesis was tested by investigating the epidemiology of malaria in frontier farming settlements in Acre State, where the most heavily exposed people were recent migrants from malaria-free areas in South and Southeast Brazil. These investigations found that 67% of the *P. vivax* and 76% of the *P. falciparum* infections in these settings were subclinical, usually with very low parasite density,^39^^,^^40^ consistent with some degree of antiparasite and antidisease immunity; few asymptomatic infections left untreated developed into overt disease over the next weeks of follow-up.^41^ The *P. vivax* accounted for 80% of infections and, somewhat surprisingly given the relatively low malaria transmission intensity, displayed extensive genetic diversity over time and space.^42^^,^^43^

**Figure 1.**
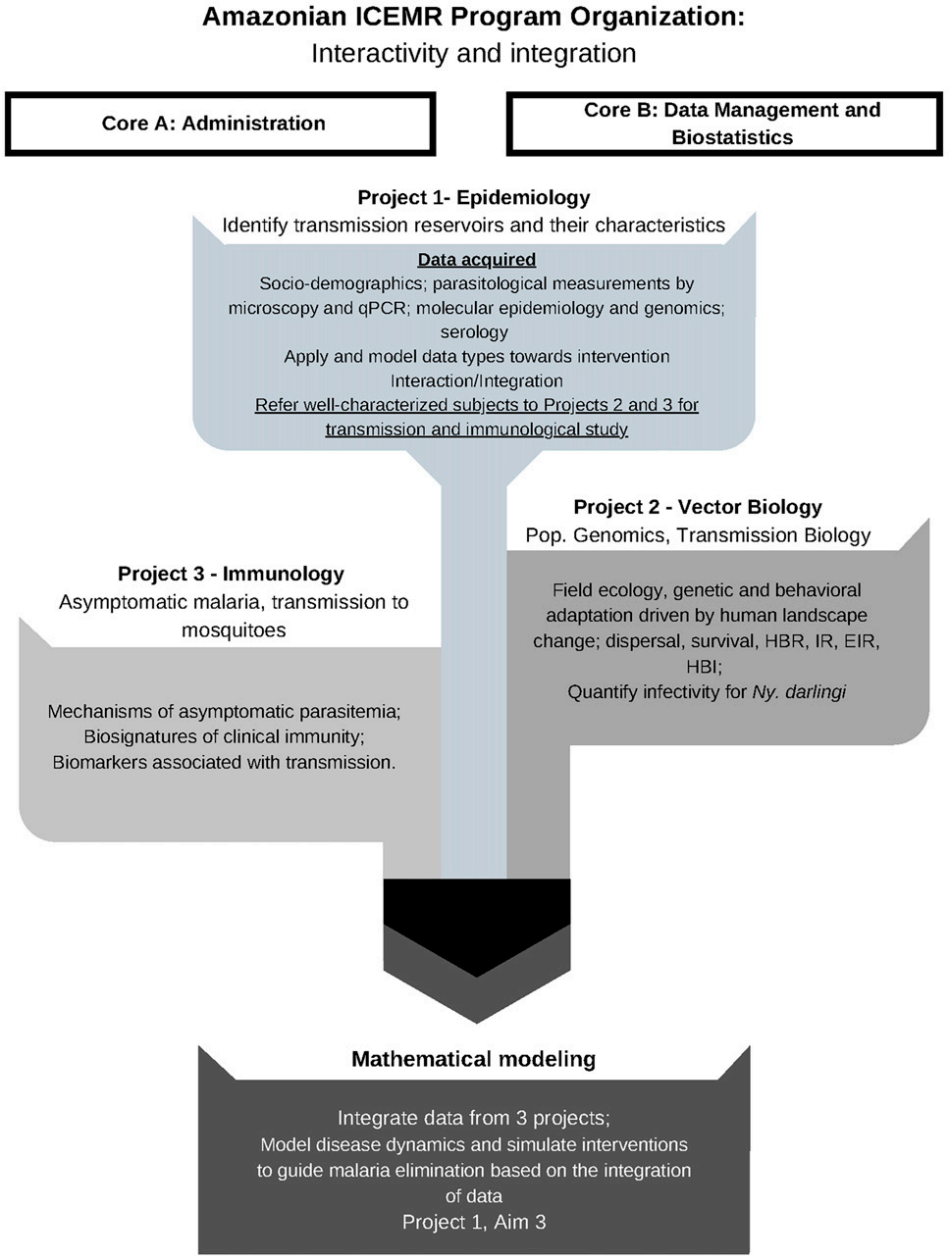
Amazonian International Center of Excellence in Malaria Research (ICEMR) Program Organization. The Amazonian ICEMR focuses on three approaches to understanding malaria transmission. The project seeks to comprehend malaria epidemiology and diagnostics in highly heterogeneous sites in the Amazon (Project 1), vector biology, ecology and genetics of local vectors (Project 2), and the transmission biology, clinical pathogenesis, and asymptomatic malaria immunology (Project 3). The integration from these projects grants the basis for mathematical modeling to understand the disease dynamics and design effective public health interventions for malaria control and elimination. The ICEMR receives support from Core A (Administration) and Core B (Data Management and Biostatistics). This figure appears in color at www.ajtmh.org.

The overall research goal of the Amazonian ICEMR is to take an integrated, comprehensive approach to understand complex sociodemographic and biological features that drive endemic malaria in the Amazon, that is, to identify human reservoirs of malaria transmission that lead to continued malaria endemicity despite standard control measures. The ultimate goal is to use such integrated information toward the regional control and elimination of *P. falciparum* and *P. vivax*. A key premise is that areas of low to moderate malaria transmission will become more common as overall malaria transmission is reduced. Thus, the Amazonian ICEMR has focused on generating data applicable to other malaria-endemic regions, where elimination is on the horizon. The Amazonian ICEMR has been structured to achieve synergy along multidisciplinary lines toward this goal (Figure 1).

**Figure 2.**
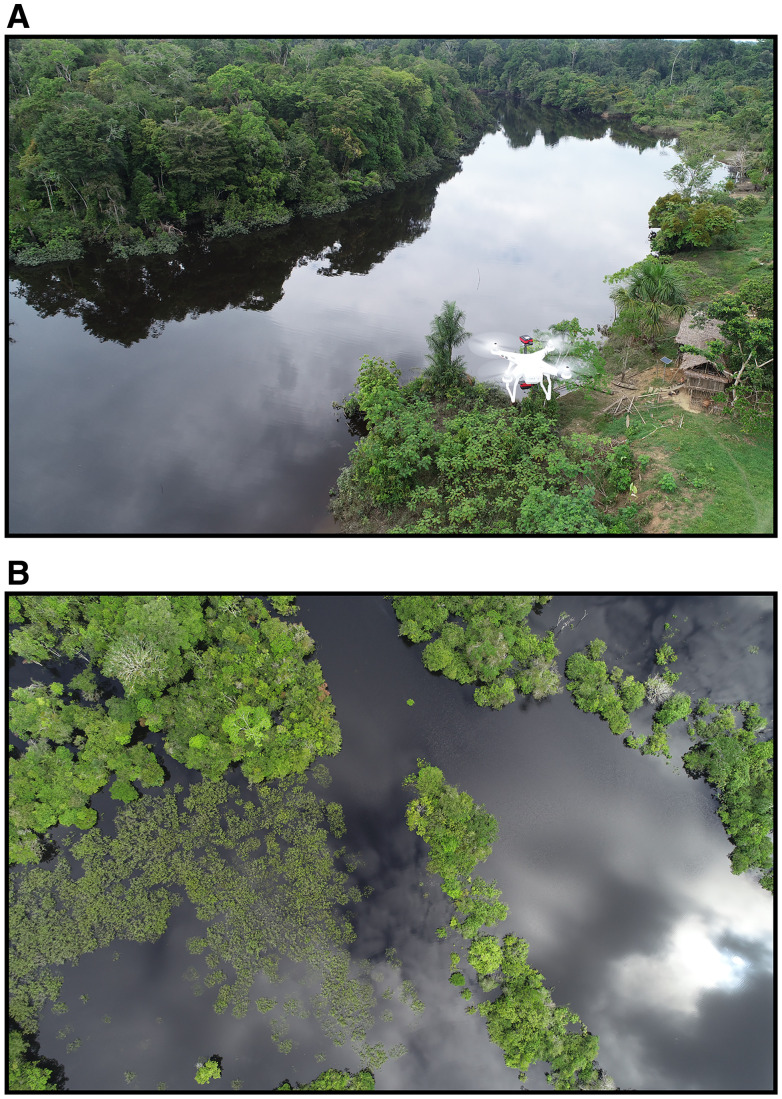
Drone imagery for the detection of *Nyssorhynchus darlingi* breeding sites. In recent years, unnamed aerial vehicles (UAVs) have become feasible tools for vector disease monitoring. (**A**) An UAV or drone employing multispectral cameras to collect aerial images from a rural riverine community in the Peruvian Amazon. (**B**) An image of a flooded area located in the Santa Rita village, within the district of Iquitos in the Amazonian region of Loreto, Peru. Later on, image analysis will be used to determine the presence and location of possible *Ny. darlingi* breeding sites.^99^ This figure is open access and permission to reuse it is through Creative Commons.

Areas with low to moderate transmission have biological and clinical complexities hidden by low morbidity and mortality: 1) patients infected by either of the two major parasite species in the region—*P. falciparum*, *P. vivax*—exhibit a spectrum of disease that includes asymptomatic and often submicroscopic malaria; 2) a high prevalence of asymptomatic and submicroscopic infections indicates that clinical immunity develops even when exposure is low; 3) disease dynamics are driven by groups that disproportionally contribute to transmission due to factors such as gender, age, and economic activity; and 4) a complex composition of malaria vectors may not be equally susceptible to patients with low to submicroscopic parasitemia. In such a context, areas with low to moderate transmission, particularly those with *P. vivax*, are a challenge to sustain elimination.

The Amazonian ICEMR has focused primarily on *P. vivax* because it is a more common cause of malaria in the region. However, given recent developments in Brazil and Peru, including the cross-border situation between Brazil and Venezuela, *P. falciparum* malaria also became a focus of the Amazonian ICEMR’s work^1^^–^^3^^,^^16^^,^^44^^–^^46^ In Brazil, the national malaria control program made *P. falciparum* a primary focus of elimination efforts in 2015.^47^ A major hotspot of *P. falciparum* malaria in Brazil is the Juruá Valley, our primary study site in Western Brazilian Amazonia. Achieving such a goal is challenged by socially determined human mobility as in the Guyana Shield, including the situation in Venezuela, driven by a challenging political situation. In Peru, after the cessation of Global Fund-supported malaria control efforts in 2010, *P. falciparum* has reemerged as a significant public health threat.^4^ The Amazonian ICEMR’s recent meetings with the Loreto Ministry of Health witnessed the high level of concern about malaria reemergence and the Ministry’s interest in harnessing the Amazonian ICEMR’s research strengths to address this emergent and timely issue. Overall, it is anticipated that the lessons learned in Amazonia will be generalizable and instructive for global malaria eradication’s audacious goal.

The Amazonian ICEMR’s basic approach is to carry out population-based longitudinal cohort studies in geographically and epidemiologically distinct sites Brazil and Peru.^25^^,^^48^ In Brazilian and Peruvian Amazonia, there are complex patterns of malaria transmission in heterogeneous and epidemiologically contrasting sites (e.g., increasing transmission versus disappearing malaria, as we have described^48^). The primary hypothesis that integrates the projects comprising the Amazonian ICEMR is that asymptomatic, submicroscopic parasitemia drives ongoing hypoendemic malaria. Residual malaria due to outdoor-biting *N*. *darlingi* mosquitoes is likely related to anthropogenically driven changing vector behaviors and genetics.^25^ Emerging, complex patterns of malaria reintroductions have made studying alternative approaches to malaria elimination critical. The ecology of the major malaria vector in Amazonia—*N. darlingi*—interacting with human behavior determines hypoendemic patterns of malaria incidence and prevalence.^49^^,^^50^ At the population-level, acquisition of nonsterilizing antidisease immunity implies that low parasitemia has the potential to maintain transmission in the endemic setting, implying a mechanism for so-called “resilient malaria.”^51^ Understanding such immunity mechanisms is central to new approaches to malaria vaccine development.^32^^,^^52^ The Amazonian ICEMR is quantifying malaria transmission from asymptomatic individuals to colonized *N. darlingi* and placing the infectivity potential of such individuals to mosquitoes in the context of immune-biomarkers of transmission.^53^

In this article, we will describe the overall hypothesis, rationale, approaches, and outcomes of the Amazonian ICEMR. The main goal is to understand patterns and determinants of two types of contrasting malaria epidemiological settings in the Amazon: residual malaria with continuing hypoendemicity, and foci of high transmission. These settings have different local ecologies (riverine, highway, and urban areas) and human behavior (e.g., levels of bednet use, different occupations, and degrees of mobility). Project 1, which focuses on the epidemiology of malaria, has three aims. The first aim comprehensively calculates and interprets local transmission indices and identify local determinants of malaria transmission. Aim 1 integrates all three Projects of the ICEMR by identifying and characterizing the context of malaria cases and referring symptomatic and asymptomatic patients to Project 2 to guide mosquito population characterization and transmission biology studies and to Project 3 for immunological experiments, respectively (Figure 1). In Aim 2, comprehensive molecular epidemiological approaches and population genetics are being used to identify temporal population changes in *P. vivax* and *P. falciparum*, detect reintroductions and parasite population replacements, and estimate parasite population complexity at baseline and potentially after interventions. Aim 3, also integrating all three Amazonian ICEMR projects, models malaria transmission dynamics, simulates the optimal intervention packages to reduce malaria in epidemiologically contrasting settings, explicitly accounting for ecological heterogeneity and differences in human sociodemographics. This Project has already contributed new solutions to ongoing and emerging malaria challenges in Amazonia. The overall ICEMR program integrates a comprehensive molecular and epidemiological data sets from Project 1 with studies of vector ecology and transmission biology in Project 2, and with laboratory-based immunology studies of asymptomatic malaria in Project 3, to provide a roadmap for new approaches to malaria elimination.

## EPIDEMIOLOGY OF ASYMPTOMATIC *PLASMODIUM* PARASITEMIA

Over the past decade, the Amazonian ICEMR has systematically investigated malaria transmission patterns in diverse settings. These include numerous riverine villages,^7^^,^^51^^,^^54^^,^^55^ frontier farming settlements,^48^^,^^56^ and urban spaces.^32^^,^^57^^,^^58^ Recently, we started pilot studies in Venezuela as it is a source of cross-border malaria in Northern Brazil.^16^^,^^59^^,^^60^

Each study site in Peru and Brazil was found to have a high prevalence of asymptomatic *Plasmodium* infections that differed in character and magnitude in sites with different geographic and sociodemographic characteristics.^7^^,^^14^^,^^30^^,^^50^^,^^61^ This finding challenges the traditional belief prevalent until the 1990s that populations exposed to relatively low forces of infection rarely develop clinical immunity.^37^^,^^38^^,^^62^^,^^63^ We are also learning from the unfortunate unique situation of Southern Venezuela that the malaria situation is indeed fluid. Based on preliminary data emerging from the region, when transmission gets out of control, clinical profiles seems to revert to more severe clinical presentations, including *P. vivax* with an unusually high proportion of cases with at least one criterion of severe malaria, as was found in Venezuela.^7^

A high malaria burden of asymptomatic and submicroscopic infections in Amazonia has consistently been found. Those infections with very low parasitemia (more than 50% of infections have < 10 parasites/µL) are heterogeneously distributed in the Peruvian communities challenging the use of microscopy during the active case detections performed by the Ministry of Health.^8^^,^^9^ In the unusual malaria situation in Venezuela, we also found that mixed infections (*P. vivax* and *P. falciparum*) are frequently underreported by microscopy.^10^^,^^40^

Conditions associated with the high prevalence of asymptomatic parasitemia are shaped by many factors, including age, time lived in the community, and occupational activities.^8^^,^^30^ As such clusters of infected individuals, symptomatic or asymptomatic, emerge as an important factor as usually is linked to specific age groups with occupations that require mobility across endemic areas.

How low-level malaria transmission reconciles with naturally acquired immunity to infection and disease remains uncertain, but malaria risk heterogeneity may provide a clue. Over time, the distribution of clinical malaria episodes experienced by each individual tends to be overdispersed: most people experience few, if any, episodes, while some individuals living in the same community are repeatedly infected.^57^ Mathematical modeling was carried out, with the basic assumption that the population is comprised of high-risk and low-risk components. The model that best fits the observed data (age-related malaria incidence and number of episodes per person over time) estimates that ∼20% of the population contributes disproportionately to overall malaria burden.^64^ One conclusion from this modeling is that individuals in the high-incidence group experience enough repeated infections to develop clinical immunity and constitute an asymptomatic parasite reservoir.^64^

Although most partially immune carriers harbor low parasite burdens, often missed by conventional microscopy, some can still infect mosquito vectors.^22^^,^^32^^,^^65^ Very few asymptomatic infections that are left untreated will eventually progress to clinical disease and become detectable by malaria surveillance.^11^^,^^12^ As asymptomatic infections tend to cluster around malaria cases detected by passive surveillance,^13^ reactive case detection may be an efficient way of detecting additional infections that are missed by routine case finding.

## HUMAN MOBILITY AND MALARIA RISK

A hypothesis that the Amazonian ICEMR seeks to test is whether mobile high-risk groups of individuals disproportionally drive malaria transmission, including asymptomatic-subclinical infections. Our premise is that by integrating parasite genetic and genomic data linked to epidemiological data, we can characterize malaria risk and characterize the factors that could make malaria resilient to interventions. We usually summarize this broad goal as “human mobility” because it builds on the drivers of human movements in contexts that facilitate the dispersion of parasites by asymptomatic infected individuals. The Amazonian ICEMR has participated in global *P. falciparum*^66^ and *P. vivax*^67^ genome projects that have assessed parasite diversity, which is key for developing tools for forensic approaches to determining the relationship of mobility and origins of introduction and reintroduction of parasites in the era of malaria elimination.

It is worth noticing that traditional population genetics metrics offer limited information if epidemiological data is absent. Metrics, such as gene flow, for example, could be an average of several transmission seasons^68^ rather than a description of recent events that are the ones we require to assess epidemiological changes. Indeed, the Amazonian ICEMR and others have used those to explain overall genetic differentiation patterns essential to obtaining a big picture of malaria in the region.^69^^,^^70^ However, we need to identify patterns to address epidemiological questions pertinent to malaria control. Unveiling such patterns require combining parasite genetics and longitudinal epidemiological data.

Human mobility has been previously linked to malaria transmission in the Peruvian Amazon. For instance, periurban villages along the Iquitos–Nauta road present parasite populations genetically very similar to those in Iquitos city, the most developed urban center of the region, suggesting that the movement for economic reasons promotes the introduction of parasites from Iquitos into these communities.^14^ Likewise, rural villages from Loreto Department, the region most struck by malaria in Peru, share genetically similar parasite populations, suggesting that mobile individuals allow for the reintroduction of parasites in distant (> 30 km) rural riverine communities.^15^

A particular example is human mobility driven by extractive economic activities, such as gold mining and logging, which involve parasite translocation by humans and aggregation of individuals in impoverished settlements that facilitate transmission. It has been hypothesized that such occupational-driven mobility patterns may generate malaria transmission corridors.^71^ Under such a model, malaria transmission may be sustained by human mobility across communities. Consistent with this process, we have documented that gold mining is an important driver of malaria crises in Venezuela^3^^,^^10^ and Peru,^16^ and logging in the Southeastern region of the Peruvian Amazon.^17^ Mobility is also important in the context of mutations associated with antimalarial drug resistance in *P. falciparum* because mining areas have multidrug-resistant genotypes, fortunately, not yet with mutations linked to the delayed clearance of ACTs.^10^^,^^18^

In recent years, new ways to measure human mobility have been developed. The use of a digital platform (GeoODK) to collect self-reported travel trajectories seems promising in assessing routes following riverine pathways to nearby villages and rural settlements, where logging, hunting, or fishing activities are carried out and suggests high connectivity among the communities in this region.^19^ We have shown that fine-scale monitoring of human movement by GPS devices in a population subset from malaria transmission areas has shown that malaria-positive participants move to nearby villages and supports the idea of approaching these communities not individually but as a network of connected units.^20^^,^^72^ Although rural villagers in the Peruvian Amazon are open to initiatives that seek to understand and eradicate malaria transmission, assessing movement through GPS devices for an entire population (∼200 or more participants) is a technical and economic challenge because such studies would require a large number of devices adapted to the rural conditions surrounding malaria transmission.^72^ Yet, experience in other infectious diseases has shown that the fine-scale tracking of mobile individuals allows for understanding heterogeneity of vector exposure, an aspect less explored in malaria transmission that undoubtedly will shed light onto patterns of residual malaria transmission.^21^

As studied by ICEMR investigators and other, illegal gold mining has been regularly associated with malaria outbreaks in Peru,^13^^,^^14^ Brazil,^16^ and elsewhere in South America,^46^^,^^59^^,^^60^^,^^73^^,^^74^ associated with gold miner occupation-related mobility as one epidemiological feature both in terms of malaria-naïve individuals arriving to an area of malaria transmission as well as infected gold miners bringing malaria away from such sites of transmission.^16^^,^^46^^,^^59^^,^^60^^,^^73^^,^^74^

Often perceived as an exclusively rural disease, malaria has been increasingly diagnosed within and near urban centers in the Amazon. Human mobility in our field site in Juruá Valley, Northwestern Brazil, favors the spread of malaria parasites across the urban-rural interface and places urban residents at increased risk of infection. They often engage in seasonal farming in high-transmission areas surrounding the cities and towns, and many maintain both urban and rural residences.^75^ Natural and human-made larval habitats—including fish farming ponds—are increasingly abundant in cities and towns and favor vector proliferation in densely populated areas, occasionally leading to outbreaks.^75^

Molecular genotyping data are consistent with sustained urban malaria transmission in the Juruá Valley region of Brazil, with a single genetic cluster comprising 32% of all of *P. vivax* infections examined over 1 year.^76^ Importantly, locally circulating *P. vivax* lineages appear to seed regional malaria transmission, as they share recent genome-wide ancestry with parasites at large geographic distances.^77^

Thus, the emerging pattern from our investigations is that the impact of mobility is modulated by its contexts. One of those factors is the parasite’s biology, which contributes to transmission maintenance in these communities. In particular, there is a high prevalence of subclinical infections with gametocytes, that is, 67% of *P. vivax* and all *P. falciparum* gametocyte carriers detected were asymptomatic and/or submicroscopic.^48^ Considering that asymptomatic infections are not detected in passive surveillance, the effect of mobility of such infected individuals is difficult to assess in traditional epidemiological investigations. Furthermore, epidemiological data usually cannot distinguish among a cluster of cases resulting from a recent introduction from an outbreak from asymptomatic patients’ ongoing transmission that was not previously detected. Such distinction is important during the elimination phase and only can be achieved by integrating genotyping with epidemiological data.

The second important factor relates to the ecological context where human populations move. Malaria in the Juruá Valley^75^ illustrates how the dynamic of vectors modulates the impact of human mobility on local transmission. Likewise, at a different geographic scale, preliminary studies in the case of migrants in the North of Brazil support the notion that vectors modulate the effect of migrants on local transmission.^78^ In particular, areas that receive a significant influx of migrants have many imported malaria cases, but that does not translate into a spike of local cases following the massive introduction of infected individuals simply because the local vector is not very efficient at breeding in proximity to those infected individuals. Thus, assessing the impact of human mobility on regional malaria resilience requires the novel integration of parasite, epidemiological, and entomological data.^72^

## VECTOR BIOLOGY

### Ecology and population structure of *Ny. darlingi*.

The earliest entomology studies focused on *Ny. darlingi* in the Amazonian ICEMR in Loreto, Peru, confirmed that this species is the primary regional vector and the only species consistently infected by *Plasmodium*.^79^ We and others demonstrated that anthropogenic and ecological changes have favored the spread of *Ny. darlingi* through numerous river systems in Loreto, Peru.^80^^–^^82^ One of our most significant vector biology discoveries was the detection of high proportions of avian blood-meals in resting *Ny. darlingi* in riverine villages outside Iquitos. This and a subsequent study underscored the adaptability of *Ny. darlingi* and also suggested that host availability is a major player in *Ny. darlingi* feeding choice, even though this species remains primarily anthropophilic.^82^^,^^83^ We also provided evidence of greater risk of transmission by *Plasmodium*-infected *Ny. darlingi* feeding outdoors compared with indoors.^79^^,^^82^

The question of whether *N. darlingi’*s behavioral heterogeneity (exo- and endophagy, exo- and endophily, host range, biting time) has a genetic or environmental basis led to studies in three ecologically distinctive communities (heavily forested, deforested, and urban) in Acre State, Western Amazonian Brazil. Only single nucleotide polymorphisms (SNPs), but not microsatellites, detected population divergence and genetic heterogeneity at a microgeographic scale.^9^ A second study using genome-wide SNPs detected genetic markers associated with indoor versus outdoor feeding in addition to dawn versus dusk feeding time, although there was also evidence of admixture among populations.^84^ A subsequent study genotyped samples of *Ny. darlingi* from these three sites in Mâncio Lima using low coverage genomic sequencing data. For the first time, we observed a statistically significant association between: 1) biting behavior and SNP markers adjacent to cytochrome P450 CYP4H14, known to be linked to insecticide resistance and 2) between blood seeking periodicity and SNP markers adjacent to genes associated with the circadian cycle (Alvarez et al unpub.data). Together, these studies emphasize the need to incorporate local dynamics of vector populations for the most effective local interventions.

### The first *Ny. darlingi* colony.

To enable research on *Plasmodium* infections in *Ny. darlingi*, a continuous colony of *Ny. darlingi* was established in Iquitos for the first time.^31^ Subsequently, systematic production of *P. vivax* sporozoites in colonized *Ny. darlingi* mosquitoes in the Peruvian Amazon was established using *P. vivax*-infected blood derived from human.^31^ Following the launch of an ICEMR site in Porto Velho, Rondonia State, Brazil, the first free-mating colony of *Ny. darlingi* in Brazil was established.^33^

### *Ny. darlingi* genomics.

Colonized *Ny. darlingi* from Iquitos, Peru were used to produce a new whole genome sequence expected to be released in 2022. It is anticipated that a newly assembled genome, as compared with previous more fragmentary genome information,^85^ will permit discovery of significant nucleotide diversity (high genetic polymorphism) linked to the rapid evolution and adaptation of *Ny. darlingi*. Anticipating evolutionary responses to increased anthropogenic and climate change, and how to more effectively control *Ny. darlingi* will require an understanding of the enormous genetic diversity that fuels it.^86^

### Nomenclature of *Ny. darlingi* and additional species detected.

A molecular phylogenetic study resulted in the proposed recognition of several new Latin American genera, especially *Nyssorhynchus* and *Kerteszia*^87^ that comprise many malaria vectors and are considered to be subgenera.^88^ This provisional nomenclatural shift is not without controversy, and a discussion is ongoing and likely to continue until whole genomes among many anopheline taxa are compared phylogenetically.^89^^–^^91^

We also detected additional anopheline vector species and distribution data were expanded. *Nyssorhynchus dunhami* was identified molecularly in Peru for the first time, in several villages South of Iquitos.^92^ Also, the sole evidence of *Ny. dunhami* infected with *P. falciparum* and *P. vivax* throughout its distribution in Brazil and Peru was recorded in Lupuna, although this species appears to have a minor role in malaria transmission.^92^ The known distribution of *Ny. benarrochi B* in malaria-endemic regions was expanded to include Madre de Dios Department in Southern Peru,^93^ Andoas District in the Datem del Maranon Province in Northern Peru, and the Amazonian Provinces of Orellana and Morona Santiago in Ecuador.^94^ This is an important finding because this species is a secondary vector throughout most of its distribution.^94^^–^^96^

### The use of drones to identify *Ny. darlingi* larval habitats.

The potential contribution of larval source management (LSM) as part of an integrated malaria control program depends on the ecology of *Ny. darlingi*.^97^ In Loreto, a study examined environmental characteristics of larval habitats relative to spatial heterogeneity of human malaria transmission and found that *Ny. darlingi* was significantly associated with low-light conditions, recent deforestation, low-vegetation index, and other anopheline species. Houses with more reported malaria cases were located nearer to *Ny. darlingi* larval habitats; targeted control of these sites would likely reduce malaria risk.^98^ In a proof-of-concept paper, these larval site characteristics together with high resolution (∼0.02 m/pixel) multispectral imagery were used to discriminate a profile of water bodies, where *Ny*. *darlingi* was most likely to breed (86.7–97% accuracy) in the Mazan District, Loreto, Peru (Figure 2).^99^

Fishponds are common in and around the town of Mâncio Lima, Western Amazonian Brazil, contributing substantially to malaria transmission. Researchers found that fishponds in rural but not urban sites appear to maintain populations of *Ny. darlingi* during the dry season, and fishponds with abundant *Ny. darlingi* larvae were those significantly associated with emergent aquatic vegetation that were actively in use.^49^ Biological larvicide application in this situation could have an impact in reducing malaria, as recently demonstrated.^100^

### Experimental *Ny. darlingi* infection: Relationship of parasitemia to mosquito infection.

Information remains scant on infectivity of *P. vivax* to mosquitoes in diverse ecological transmission contexts, which led us to measure the transmissibility of clinical and subclinical *P. vivax* malaria parasite carriers to the major mosquito vector in the Amazon Basin, *Ny. darlingi* using membrane mosquito feeding assays (MFA), in which blood from an infected individual is placed into a membrane feeder and offered to mosquitoes.^31^^,^^33^^,^^34^^,^^101^

In Brazil, 15 asymptomatic individuals with positive PCR in the same blood sample used for MFA, eight were able to infect mosquitoes, as evidenced by the oocysts found in their midgut ranging from one to seven per midgut. Importantly, even with undetectable parasitemia by qPCR, asymptomatic contribute with low rates of transmission to anophelines, suggesting their potential role in sustaining the *P. vivax* cycle in hypoendemic areas.^32^

In Peru, none of the asymptomatic low-density PCR detected infections infected a single mosquito suggesting that additional assessment to determine the infectivity of low-level parasitemia in the context of the Peruvian Amazon is needed.^65^ However, our results are consistent with studies showing that *P. vivax* gametocyte and parasite density in symptomatic individuals are closely related to mosquito infectivity.^65^

Paradoxically, the lack of a linear relationship of absolute parasitemia and gametocytemia to infectivity of *P. vivax-*infected individuals for *Ny. darlingi* mosquitoes is a consistent finding, mirrored by other *Plasmodium*-mosquito relationships in diverse settings.^102^^–^^105^ In Project 3, we continue to explore the contribution of human host immunological factors to parasite infectivity to mosquitoes in the endemic field setting.^52^

## IMMUNOLOGY OF *PLASMODIUM VIVAX* MALARIA

Knowledge about parasite biology and mechanisms involved in the control of *P. vivax* blood-stage by the immune system remains scant and has been slowed by the difficulty of in vitro culture mainly because the infection is restricted to reticulocytes that rapidly mature into erythrocytes.^52^ An important facet of malaria is that sterile immunity is uncommon, so populations in endemic regions have recurrent infections. Two distinct hypotheses can be proposed to address this issue: 1) innate immune cells from asymptomatic patients become hyporesponsive to *Plasmodium* stimulation preventing systemic inflammation, leading to impaired acquired immunity, which is inefficient in controlling infection and allowing parasite transmission; and 2) asymptomatic patients develop robust acquired immunity that maintains low parasite biomass preventing systemic inflammation and vector infectivity. The pathobiology pathogenesis of malaria is complex and the immune system has to kill the parasite and avoid tissue damage simultaneously.^106^ In this context, it has to be determined whether there are specific patterns of the immune response associated with the development of symptomatic or asymptomatic persons.

**Figure 3.**
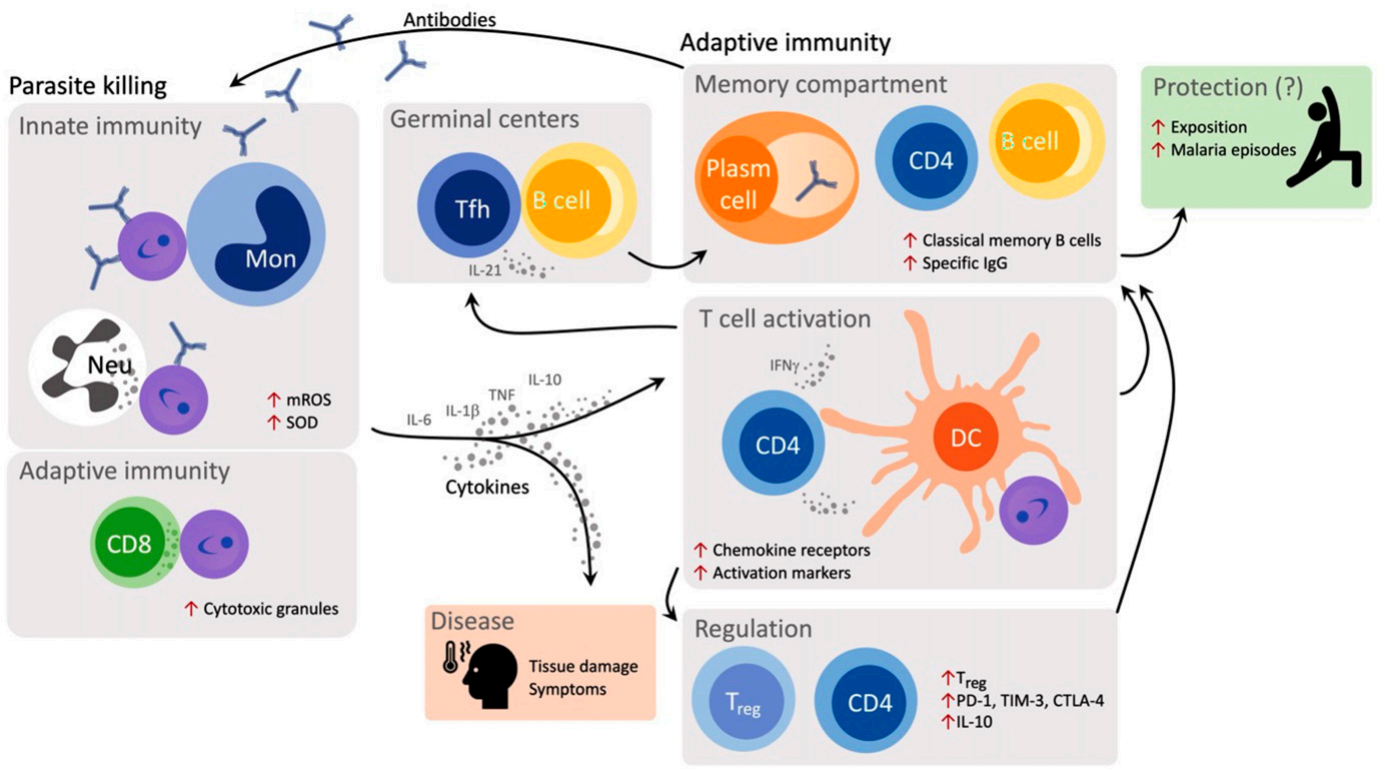
Immune response against *Plasmodium vivax* infection. Monocytes, neutrophils, and CD8^+^ T cells mediate parasite killing through production of mitochondrial reactive oxygen species (mROS), SOD, and cytotoxic granules, respectively. Parasite killing by monocyte is antibody dependent. Cytokines produced by innate cells and act on cells from adaptive immune response, shaping effector functions of CD4^+^ T helper cells and Treg. Parasite burden and cytokines are associated with symptoms and trigger the expression of chemokines, chemokine receptors and activation and inhibitory molecules. The production of IFN-gamma is probably affected by this environment. The IL-21 is also induced by *P. vivax* infection, leading to T follicular helper (Tfh) expansion and it is associated with antibody production by plasma cells. The number of malaria episodes are associated with increased frequencies of Tfh cells and classical memory B cells, probably impacting protection.

We have directed our efforts toward identifying immune-biomarkers of resistance and susceptibility to disease to better understand malaria pathogenesis (Figure 3). It is established that the paroxysm triggered by inflammatory cytokines is a clinical hallmark of acute malaria and these molecules have been associated with both control and symptoms of the disease. Monocytes, the primary source of proinflammatory cytokines in the circulatory system, are expanded during acute *P. vivax* infection.^107^ Classical and patrolling monocytes produce large amounts of IL-1β after stimulation with lipopolysaccharide (LPS; endotoxin), probably due to the NLRP12/NLRP3-dependent activation of caspase-1.^108^ A third subset, the intermediate monocytes, have an increased ability to phagocytose parasite-infected reticulocytes and to produce intracellular reactive oxygen species production. Recent data from our group suggested that *P. vivax* infection fosters a metabolic shift favoring the production of mitochondrial reactive oxygen species (mROS) by monocytes. Monocytes from malaria patients are reprogrammed to maintain their effector functions increasing their glycolysis rate and decreasing the production of ATP via oxidative phosphorylation.^109^ With this shift, mROS is produced as a result of the energy derived from the variation in mitochondrial membrane potential generated by electron transport via the electron transport chain. The close contact of mitochondria with phagolysosomes containing the *P. vivax*-infected reticulocytes suggests the involvement of mROS with parasite killing. In parallel, CD8^+^ T cells are also involved in parasite killing during acute malaria. CD8^+^ T cells express large amounts of cytotoxic proteins and form immunological synapses with *P. vivax*-infected reticulocytes. Consequently, CD8^+^ T cells kill intracellular parasites and infected host cells, which lose cholesterol from their membranes becoming susceptible to granulysin during infection.^110^

The inflammatory environment resulting from the innate immune system activation impacts the development of the adaptive response. High levels of cytokines have been associated with the expression of programmed death-1 (PD-1) and its ligand, limiting T-cell-effector function. Our studies show that *P. vivax* infection leads to increased expression of inhibitory molecules, such as PD-1 cytotoxic T lymphocyte attenuator-4 (CTLA-4) and T cell immunoglobulin domain and mucin domain-3 (TIM-3) on T cells. These lymphocytes retain the ability to respond to antigens and the cytokine response is reestablished when CTLA-4, PD-1, and TIM-3 are contemporaneously blocked, indicating that induction of multiple regulatory molecules during *P. vivax* infection is necessary to impair T cell function.^110^ The expression of PD-1 is also associated with diminished function of regulatory T cells in *P. vivax*-infected patients. PD-1 expressed Treg display inflammatory characteristics and were less capable of suppressing proliferation of CD4^+^ T cells. Importantly, the presence of these regulatory T cells was correlated with augmented levels of bilirubin in malaria patients.^110^ This finding reflects similar findings with Tregs in *P. falciparum* malaria in Peru.^111^

Studies have been focused on the importance of humoral immunity to malaria and immunoglobulin production against *Plasmodium* is dependent on T cells. More specifically, *P. vivax* infection triggers the expansion of T follicular helper (Tfh) cells, which favors the generation of plasma cells that secrete high-affinity antibodies and maintain long-lived memory B cells. Although the expression of PD-1 on T cells during malaria suggests impaired function of some cell subsets, PD-1 expression, in association with the chemokines, CXCR5 and ICOS, specifically defines circulating counterpart of *bona fide* Tfh cells. Importantly, reinfection with *P. vivax* further expands Tfh cells, which is associated with increased frequencies of classical memory B cells and levels of IgG.^112^

The conceptual understandings from studies on the immunopathology of symptomatic malaria paved the way to further explore mechanisms underlying asymptomatic malaria (Table 1). Since 2018, an endemic urban area for malaria due to *P. vivax* in the Brazilian Amazon is screened to identify asymptomatic cases. Distinct from what is observed during symptomatic malaria, there are no alterations in the common hematological and biochemical biomarkers supporting the lack of systemic inflammation in asymptomatic individuals.^32^ Asymptomatic carriers of *P. vivax* display measurable antibody levels against *P. vivax*, although with lower levels of IgG compared than symptomatic patients. Those lower levels suggest the hypotheses that there could be a reduction of antigen-specific antibody levels or changes affinity of anti-*P. vivax* IgG over time.^52^^,^^113^^–^^115^

**Table 1:** Some immunological features of symptomatic and asymptomatic malaria*

	Symptomatic*	Asymptomatic*	References
Hematological and biochemical parameters	Some altered	Not altered	Not available
Levels of specific antibodies against *P. vivax* antigens	Increased	Increased	^32^ ^,^ ^52^
Expression of regulatory molecules on CD4^+^ and CD8^+^ T cells	Increased	?	^52^ ^,^ ^111^
Frequency of circulating CD4^+^ T cells	Decreased	?	^52^
Frequency of circulating CD8^+^ T cells	Decreased	?	^52^
Frequency of monocytes	Increased	?	^52^ ^,^ ^107^ ^,^ ^110^
Frequency of follicular helper T cells	Increased	?	^32^ ^,^ ^52^ ^,^ ^112^
Frequency of regulatory T cells	Increased	?	^52^ ^,^ ^111^
Leukocyte response to innate stimulus	Increased	?	^52^
Serum cytokines	Increased	?	^52^ ^,^ ^53^

* Compared with healthy controls or patients after treatment. Note on data sources: References as indicated or unpublished observations/data from Amazonian ICEMR.

These findings support the need for deep experimental investigations of immune mechanisms in asymptomatically infected individuals. We are currently carrying out extensive phenotyping of circulating leukocytes and performing functional assays to identify mechanisms that explain the lack of systemic inflammation, the absence of symptoms, and the lower parasitemia observed in asymptomatic individuals. The project provides resources to study these hypotheses and also contribute to the education of the community on malaria, fostering the awareness of the local health authorities of the occurrence of undiagnosed malaria cases.

Key antigenic targets of naturally acquired antibody-mediated immunity to *P. vivax* malaria remain to be determined and cohort studies may offer some useful insights. We have focused on antibodies to the cysteine-rich domain II of *P. vivax* Duffy binding protein (PvDBP) that inhibit binding of this parasite ligand to its receptor on red blood cells, the Duffy antigen/receptor for chemokines (DARC), known as binding-inhibitory antibodies (BIAbs). We showed that high levels of BIAbs are associated with a > 40% decrease in the prospective risk of clinical vivax malaria in subjects.^115^ Importantly, human monoclonal antibodies with binding-inhibitory properties partially inhibit ex-vivo red blood cell invasion by *P. vivax* merozoites and target a conserved PvDBP epitope.^113^^,^^114^

Using genome-level protein microarrays in which a large number of *P. falciparum* and *P. vivax* asexual and sexual stage proteins identified from proteomic and gene expression profiling are put onto a chip for probing with sera from human subjects,^116^ a limited set of *P. falciparum* protein antigens was associated with the development of naturally acquired clinical immunity in the Peruvian Amazon.^117^ Similarly, *P. vivax* antigen relapse was distinguished from reinfection by a merozoite surface protein, MSP10, as the top hit among other proteins.^118^ These data identified candidates for seroepidemiological tools to support malaria elimination efforts in *P. falciparum-* and *P. vivax-*endemic regions.

In this context, we focused on the production and use of recombinant Merozoite Surface Proteins for *P. vivax* (PvMSP8 and PvMSP10) and *P. falciparum* (PfMSP10 and PfRH2b) as serological markers (SEM) of recent exposure in low-to-moderate transmission settings in coendemic areas of the Peruvian Amazon region.^24^^,^^119^^–^^121^ In addition, using a panel of 34 SEM for *P. vivax* in cohorts from Peru (Lupuna and Cahuide), Brazil, and Thailand, we found a strong correlation of high IgG levels against this SEM with age; but living in Lupuna and being male were associated with 20 and 15 SEM, respectively, indicating the high exposure in this community. The performance of these 34 SEM to classify recent exposure was lower in Peru than in Thailand and Brazil; this could be due to differences in malaria transmission intensity.^121^ Future prospects in this area are the study of IgM antibody response and IgG subtypes against these seromarkers their functional characterization.^122^

## CONCLUSION

Malaria in Amazonia presents complexities hidden by its low levels of morbidity and mortality. The Peruvian MZP has been designed based on data from the Amazonian ICEMR to accelerate malaria control and elimination in the region.^123^ People infected by either *P. vivax* or *P. falciparum* exhibit a broad spectrum of disease severity, including a high proportion of asymptomatic submicroscopic infections with gametocytes. Thus, those are likely to be untreated transmission reservoirs because of the lack of clinical illness that typically prompts treatment. High-risk groups linked to particular economic activities exhibit high mobility and disproportionally drive malaria transmission in various ecological contexts. Such dynamics of mobile and asymptomatic malaria makes the disease resilient to elimination. Although success can be achieved in dramatically reducing malaria morbidity and mortality by scaling up interventions, there is a need to characterize factors that make malaria resilient to optimize surveillance toward the long-term goal of containing reintroductions. Correctly modeling disease dynamics considering such factors is critical to inform malaria elimination programs. Such an understanding is required to make malaria elimination both feasible and sustainable. The mechanisms leading to clinical immunity–asymptomatic parasitemia in low-transmission areas like Amazonia may enable new vaccine development approaches and understanding of malaria pathogenesis.

## IMPACT OF COVID-19 PANDEMIC ON AMAZONIAN ICEMR RESEARCH ACTIVITIES

The impact of the COVID-19 on malaria in Amazonia has been difficult to determine precisely. The COVID-19 pandemic led to a strict lockdown imposed throughout Peru in 2020; all field and laboratory activities were stopped for 6 months. After the first wave between March and August 2020, funds had to be reallocated for humanitarian purposes to purchase personal protective equipment (PPE) as malaria research activities were impossible to carry out. Despite numerous obstacles, the ICEMR team was able to carry out remote work, which included focusing on the organization and analysis of large databases, preparation, and submission of ICEMR-related and other manuscripts. Timely experiments and small data collections were allowed under safe conditions. In 2021, activities were reactivated at better speed and planned for long-term samples collections performing the experiments and sample collections activities as usual to accomplish the committed deadlines. In this context, we were able to describe the direct impact of COVID-19 on malaria in Loreto, as evidenced by an apparent reduction in malaria control activities by the Peruvian MZP.^124^ At the time, there was substantial concern that reduction in malaria control activities might lead to a hidden increase in malaria cases. This has not yet been observed, paradoxically—and consistent with epidemiological themes of the Amazonian ICEMR—the prolonged regional shutdown of transportation during the highest malaria transmission season in 2020 that lowered occupation-related and other mobility may have in fact reduced malaria transmission. The effect of COVID-19 on malaria resurgence and excess morbidity and mortality due to malaria will be determined by continued surveillance by the Amazonian ICEMR.

No effect of COVID-19 on malaria case incidence or parasitemia prevalence was apparent in Brazil. As in Peru, COVID-19 surveillance took precedence, including in Western Brazilian Amazonia In Mâncio Lima, the Amazonian ICEMR primary study in Brazil, field studies have, since 2020, expanded to include SARS-CoV-2 antibody measurements during the ongoing COVID-19 pandemic in the hard-hit Amazon Basin of Brazil. Three consecutive cross-sectional serosurveys have been carried out (October–November 2020, April–May 2021, and October–November 2021), complemented with the genomic characterization of locally circulating SARS-CoV-2 isolates in August 2020 and April 2021. We identified possible interactions between dengue fever and COVID-19 during the first pandemic wave and tested whether the emergence of Gamma variant, which dominated the second SARS-CoV-2 transmission wave in the Amazon between December 2020 and June 2021, led to increased morbidity in the overall population of children.^125^^,^^126^ Data from the latest serosurvey are currently under analysis and will allow for estimating the duration of naturally acquired and vaccine-induced antibody responses in the study population.
